# The role of neutrophils in the pathogenesis of abdominal aortic aneurysms

**DOI:** 10.1007/s00011-026-02258-x

**Published:** 2026-04-28

**Authors:** Stephen A. Addo, Kamila Wojnar-Lason, Amritha Sreekumar, Tamasi Roy, Douglas Sloan, Gábor Csányi

**Affiliations:** 1 Vascular Biology Center, Medical College of Georgia at Augusta University, 1460 Laney Walker Blvd. CB-3213A, Augusta, GA 30912 USA; 2 Department of Pharmacology and Toxicology, Medical College of Georgia at Augusta University, 1460 Laney Walker Blvd, Augusta, GA 30912 USA

**Keywords:** Abdominal aortic aneurysm, Neutrophils, Neutrophil extracellular traps, Extracellular matrix, Neutrophil Elastase, Myeloperoxidase, NADPH oxidase

## Abstract

**Background:**

Abdominal aortic aneurysm (AAA) is a progressive degenerative vascular disease characterized by structural weakening and pathological dilatation of the abdominal aortic wall. Currently, no effective pharmacological therapies are available, and treatment remains largely limited to surgical intervention. This underscores the urgent need to better understand the mechanisms driving disease development and progression. Among the cellular mediators implicated in AAA, neutrophils have emerged as key contributors to vascular inflammation and tissue destruction.

**Methods:**

We performed a comprehensive literature review of original research articles and relevant reviews addressing the role of neutrophils in AAA pathogenesis. Studies were identified through systematic searches of major databases, including PubMed and Google Scholar, using the keywords “abdominal aortic aneurysm”, “neutrophils”, “vascular inflammation”, “oxidative stress”, “proteases”, and “extracellular matrix degradation”. Both experimental and clinical studies were included to provide an integrated overview of current knowledge in the field.

**Results:**

Accumulating evidence indicates that neutrophils actively infiltrate the aortic wall during AAA development, where they release a diverse array of effector molecules, including reactive oxygen species, proteolytic enzymes, pro-inflammatory cytokines, chemotactic mediators, and granule proteins. Collectively, these mediators sustain chronic vascular inflammation, promote extracellular matrix degradation, and contribute to progressive structural weakening of the arterial wall. This review summarizes both established and emerging roles of neutrophils in AAA pathogenesis, with a particular focus on their contribution to vascular inflammation, thereby providing a conceptual framework for future diagnostic and therapeutic development.

**Conclusion:**

Neutrophils are central regulators of AAA pathogenesis through their multifaceted roles in vascular inflammation and extracellular matrix remodeling. Targeting neutrophil activation and downstream inflammatory pathways may represent a promising therapeutic strategy. A deeper mechanistic understanding of neutrophil-driven processes may facilitate the development of novel biomarkers and pharmacological approaches aimed at limiting AAA progression and preventing rupture.

## Introduction

An aneurysm is a localized dilation of a larger blood vessel related to regional remodeling and weakening of the wall structure. Although most aneurysms develop in larger arteries, aneurysms may also form in the venous circulation. Primary aneurysms are caused by matrix defects in the vessel wall (e.g., Marfan syndrome, Loeys-Dietz syndrome, Ehlers-Danlos syndrome, and aneurysms associated with congenital bicuspid aortic valves) [1]. Secondary aneurysms develop due to extracellular matrix (ECM) degradation, chronic vascular inflammation, cell death in the vasculature, pathological arterial remodeling, and vessel wall injury [2]. Although pathological vessel wall dilations may occur throughout the entire vascular tree, aneurysms are common in some locations (e.g., thoracic and abdominal aorta, anterior cerebral artery) and exceedingly rare in other places (e.g., subclavian and radial artery) [3]. Morphologically, aneurysms are usually considered to be either fusiform or saccular. This review article focuses on abdominal aortic aneurysms (AAA), one of the most common types of aneurysms, and a potentially life-threatening condition [4].

AAAs constitute a significant public health challenge, predominantly impacting males within the age bracket of 65 to 85 years [5]. While a minority of AAA patients may exhibit nonspecific symptoms, such as lumbar or abdominal pain, most AAAs remain clinically silent until dissection or rupture. Aortic dissection is a tear in the aortic wall where blood surges between the vascular layers. Aneurysm rupture is when the vessel wall bursts, leading to internal bleeding. Both aortic dissection and rupture represent a medical emergency and are associated with a significant mortality risk. Annually, ruptured AAAs result in an estimated 15,000 deaths in the United States [6–8]. AAA ranks as the fifteenth leading cause of death in the United States [9–11]. In 2017, age-standardized abdominal aortic aneurysm mortality across the European Union (15 + countries) averaged 5.0 deaths per 100,000 men and 2.1 deaths per 100,000 women, with the highest rates in the United Kingdom (7.5 and 3.7 per 100,000, respectively) and the lowest in Portugal (2.8 per 100,000 men) and Spain (1.0 per 100,000 women) [12]. The risk of rupture increases as the outer diameter of the abdominal aorta increases, with the growth rate initially slow that may accelerate at later stages of AAA progression. The overall mortality rate after rupture is high, ranging from 65% to 85%, with the majority of fatalities occurring before patients receive surgical intervention [7, 8].

**Fig. 1:**
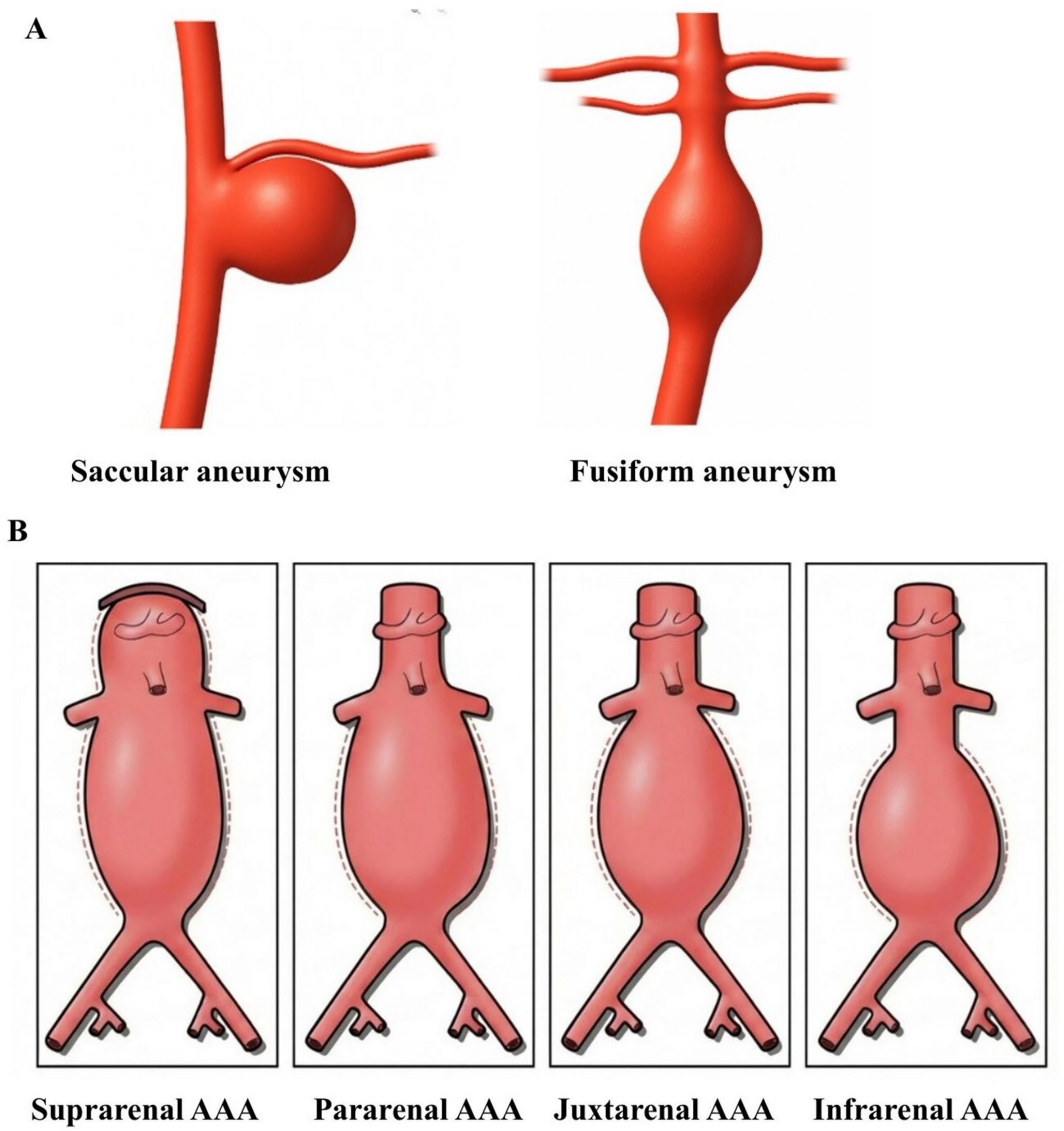
**A** Common morphological features of AAA (saccular vs. fusiform aneurysms). **B** Structural and anatomical classification of human AAA. Aneurysms are categorized based on their anatomical relationship to the renal arteries; suprarenal AAA extends above the renal arteries, often involving visceral branches, pararenal AAA extends to and involves the level of the renal artery origins, juxtarenal AAA extends to immediately below the renal arteries without an intervening normal segment, and infrarenal AAA begins below the renal arteries with a preserved proximal aortic neck

Characteristically, human AAA manifests as a localized and irreversible dilation of the abdominal aorta, with a predilection for the infrarenal segment [13]. A pathological threshold for AAA is established at 3 cm or greater in external diameter. Morphologically, saccular AAAs (asymmetric enlargement of the aorta) account for approximately 5% of all AAAs, with most being fusiform abdominal enlargements (Fig. 1A) [14, 15]. Anatomically, these are classified based on their relationship to the renal arteries, with the infrarenal type being the most common **(**Fig. 1B). Several risk factors have been implicated in the pathogenesis of AAA, including advanced age, male sex, smoking, and positive family history [16]. While risk factors for AAA, such as age, smoking, hypertension, and family history, are similar between sexes, smoking appears to confer a greater risk in women [17]. Although AAAs are less prevalent in women, they exhibit faster growth [18], a fourfold higher rupture rate during surveillance, and a threefold increase in fatal rupture compared to men [18]. Rupture also occurs at smaller diameters, likely due to women’s inherently smaller aortic dimensions [19, 20].

The current therapeutic landscape for AAA is limited to surgical interventions, which primarily aim to repair or reinforce the structurally compromised aortic wall [13, 21]. While lifestyle modifications (e.g., smoking cessation, maintaining a healthy diet, regular moderate exercise, and stress management) and pharmacotherapies targeting cardiovascular risk factors (e.g., antihypertensive, lipid-lowering, and anti-inflammatory medications) are critically important for AAA management, they do not typically halt the progression of AAA. Preclinical studies have identified numerous pharmacological targets that stabilize the aortic wall, decrease vascular inflammation, inhibit matrix remodeling, and prevent or slow down AAA development and progression [22–25]. To date, no pharmacological strategies have been proven effective at inhibiting AAA growth and preventing rupture in patients [16]. This therapeutic lacuna highlights the need for intensified basic and clinical research to identify new drug targets that can effectively modulate the fundamental cellular and molecular mechanisms underlying human AAA development, progression, and rupture.

The pathogenesis of AAA is characterized by the structural and functional deterioration of the aortic wall, leading to adverse remodeling and localized progressive dilatation of the abdominal aorta. This structural remodeling is mediated by the activation of proteolytic enzymes, leading to the degradation of ECM, apoptosis of vascular smooth muscle cells (VSMCs) in the media, recruitment of circulating inflammatory cells into the aortic wall, secretion of inflammatory cytokines, and excess reactive oxygen species (ROS) production [26]. While the molecular mechanisms underlying AAA development remain incompletely elucidated, chronic vascular inflammation is widely recognized as a pivotal driver of AAA pathology. The infiltration of circulating inflammatory cells into the aortic wall is a hallmark of AAA. Previous studies have demonstrated that neutrophils contribute to AAA pathogenesis through various mechanisms, including oxidative stress, VSMC death, proteolytic degradation and remodeling of the medial layer, adventitial inflammation, and intraluminal thrombus (ILT) formation [27]. This review aims to critically analyze previously described as well as emerging new mechanisms by which neutrophils contribute to AAA pathogenesis and discusses current knowledge on their contributions to vascular inflammation, matrix degradation, weakening, and remodeling of the aortic wall, leading to the initiation and development of AAA, and its progression to rupture.

## Neutrophil biology

**Fig. 2:**
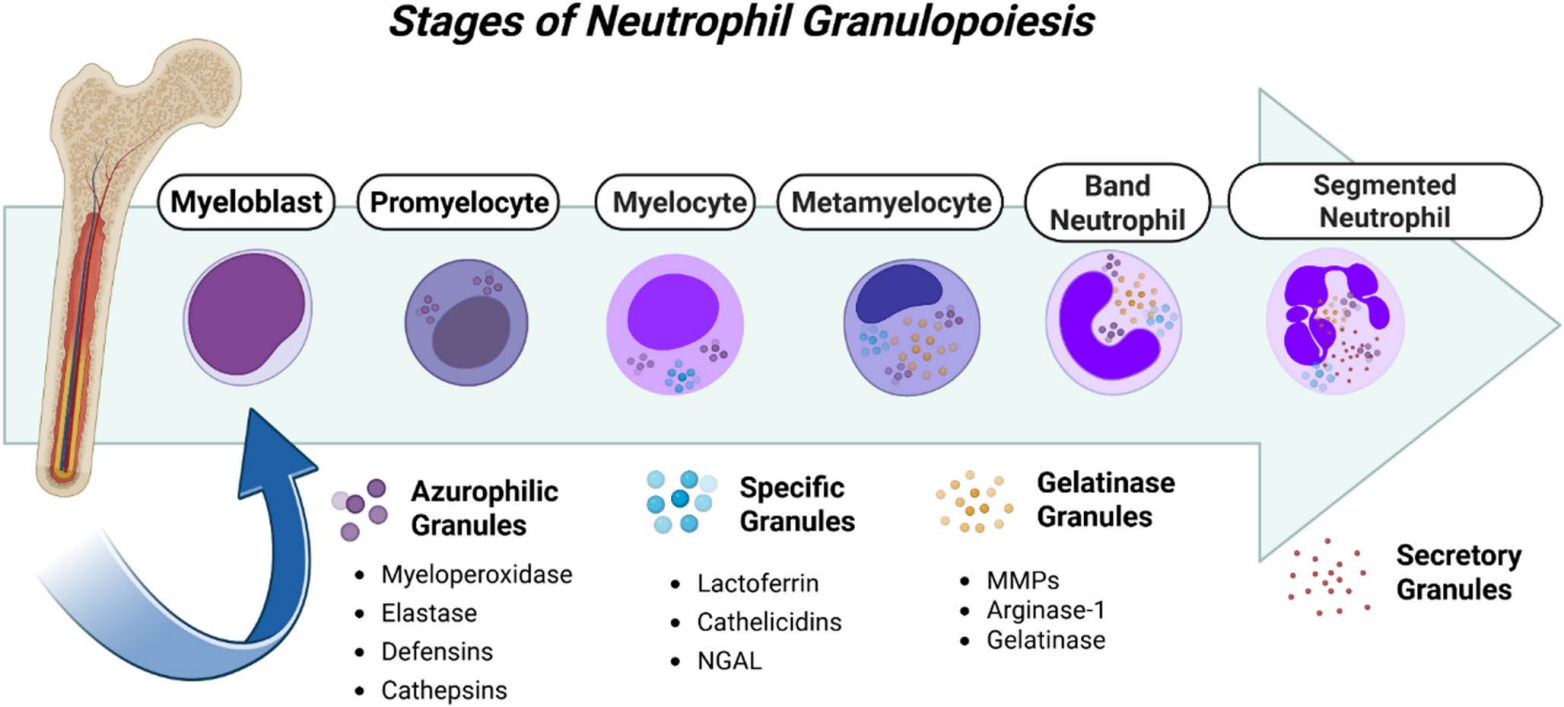
Neutrophil development occurs in the bone marrow through sequential stages: myeloblast, promyelocyte, myelocyte, metamyelocyte, band cell, and mature neutrophil. Each stage is characterized by progressive nuclear segmentation and granule formation, producing functional neutrophils ready for immune defense

Neutrophils are the most abundant leukocytes in the human bloodstream. They serve as critical effector cells within the innate immune system and act as the first line of defense against acute inflammation and microbial invasion [28]. Traditionally, neutrophils have been considered short-lived cells with an estimated circulating half-life of approximately 7 h in humans and 8–10 h in mice [29]. However, recent studies have challenged this paradigm and suggested that neutrophil lifespan may be more variable due to methodological limitations and inconsistencies in labeling techniques [30]. Neutrophils originate from hematopoietic stem cells in the bone marrow through granulopoiesis. This process is tightly regulated by colony-stimulating factor 3 (CSF3), also known as granulocyte colony-stimulating factor (G-CSF) [31]. Within this developmental pathway, granulocyte-monocyte progenitors (GMPs) undergo differentiation into mature neutrophils under the influence of G-CSF [32]. G-CSF is essential for the egress of neutrophils from bone marrow into the systemic circulation. This process is mediated through G-CSF-induced downregulation of CXCL12 and its receptor CXCR4, critical regulators of neutrophil retention within the bone marrow niche [33]. Experimental studies in mice demonstrate that genetic ablation of CSF3 or CSF3R causes severe neutropenia, while human genetic studies show that loss-of-function mutations or functional impairment of CSF3 or CSF3R likewise result in profound neutropenia, collectively establishing the CSF3–CSF3R signaling axis as essential for neutrophil production, homeostasis, and mobilization [34]. During development, neutrophils proceed through a well-defined sequence of maturation stages, progressing from myeloblasts to promyelocytes, myelocytes, metamyelocytes, band neutrophils, and finally, fully mature segmented neutrophils (Fig. 2) [35]. Each transition in neutrophil maturation is characterized by the acquisition of specific morphological and biochemical features. The progression from myeloblast to promyelocyte is marked by the emergence of primary (azurophilic) granules, which contain essential antimicrobial components such as myeloperoxidase (MPO), elastase, and defensins [36]. As differentiation advances from the myelocyte to metamyelocyte stage, secondary (specific) granules form, housing critical immune effectors such as lactoferrin and cathelicidins [37]. Finally, tertiary (gelatinase) granules emerge during the transition from band to segmented neutrophils, containing matrix metalloproteinases (MMPs) that facilitate neutrophil migration and tissue remodeling [38]. Mature neutrophils are characterized by the presence of secretory granules and the expression of surface markers, including CD16, CD15, CD66b, the integrin complex CD11b-CD18, and the complement receptors C3b/C4b [35, 39]. This orchestrated sequence of granule biogenesis endows neutrophils with a broad arsenal of antimicrobial molecules, equipping them for efficient pathogen clearance and inflammation resolution.

## Neutrophil heterogeneity

Neutrophils are traditionally viewed as a homogenous cell population and short-lived first responders in the immune system. They are now recognized as a heterogeneous and functionally plastic group of cells with diverse roles in host defense, inflammation and disease pathology [40, 41]. The concept of neutrophil heterogeneity was first introduced in 1986 with the identification of distinct neutrophil subpopulations: low-density neutrophils (LDNs) and high-density neutrophils (HDNs), characterized by density gradient centrifugation [42]. High-density neutrophils segregate with the granulocyte-rich fraction due to their higher buoyant density, whereas LDNs anomalously partition with the peripheral blood mononuclear cell (PBMC) layer, a phenomenon increasingly associated with chronic inflammatory and autoimmune conditions, suggesting functional and phenotypic divergence within the neutrophil lineage. Neutrophil heterogeneity refers to distinct neutrophil subpopulations within the overall neutrophil population, exhibiting variations in phenotype and function. For example, while some neutrophils are highly proficient in ROS, chemokine, and cytokine production to promote inflammation, others secrete pro-resolving mediators that contribute to the resolution of inflammation and restoration of normal tissue homeostasis [43]. Recent advancements in single-cell and multi-omics technologies have profoundly reshaped our understanding of neutrophil biology, revealing that distinct neutrophil subsets defined by specific surface markers, maturation states, transcriptional signatures, and functional polarization exert diverse and context-dependent roles in physiology as well as the pathogenesis of both acute and chronic vascular inflammatory diseases [44]. Immature neutrophils, predominantly retained within the bone marrow, express CD33, CD15, CD11b, CD16, and CD10 [45, 46], and are mobilized into peripheral circulation in response to chemokines through CXCR4 downregulation and CXCR2-mediated signaling (e.g., CXCL1, CXCL2, CXCL8) [47–49]. Once in circulation, mature neutrophils are identified by high expression of CD16^hi^, CXCR2^hi^, and CD62L^hi^, alongside CXCR4^low^ levels, enabling efficient endothelial adhesion and chemotaxis [50]. Upon inflammatory stimulation, neutrophils undergo activation characterized by the mobilization of vesicular contents and upregulation of surface adhesion molecules. CD11b/CD18 (Mac-1) is rapidly expressed to mediate firm adhesion to the endothelium [51]. CD66b, a marker stored in specific granules, is translocated to the plasma membrane during degranulation [52]. In patients with acute inflammatory conditions, such as trauma, infection, circulating levels of CD16^low^/CD62L^low^ neutrophils are markedly increased. In contrast, chronic inflammatory diseases, including chronic obstructive pulmonary disease and human immunodeficiency virus infection, do not significantly alter the abundance of this neutrophil subset [53].

### Neutrophil heterogeneity in cardiovascular inflammation and AAA

Although less characterized than in malignant or infectious diseases, neutrophil heterogeneity plays a significant role in cardiovascular inflammation. Aging neutrophils in circulation undergo transcriptomic and proteomic reprogramming, resulting in a hyperactive phenotype [54]. These aged neutrophils show enhanced neutrophil extracellular trap (NET) formation and increased vascular and myocardial damage capacity compared to their younger counterparts [55]. Low density granulocyte subsets with high inflammatory potential are associated with atherosclerotic burden in autoimmune diseases such as lupus and psoriasis [56, 57]. However, their role in non-autoimmune cardiovascular pathology remains poorly understood. Vafadarnejad et al. demonstrated that neutrophil heterogeneity evolves dynamically in murine models of myocardial infarction [58]. During acute post-myocardial infarction phase (day 1), immature neutrophils with transcriptional profiles like bone marrow-derived cells initially infiltrate the ischemic heart. By day 3, two distinct subsets emerged including SiglecF^low^ neutrophils resembling circulating populations and a SiglecF^hi^ subset localized to the infarcted myocardium, exhibiting enhanced phagocytic activity and ROS production. SiglecF^hi^ neutrophils were also observed in murine models of atherosclerosis, indicating a potential role in both acute and chronic vascular inflammation.

Expanding the understanding of neutrophil heterogeneity in vascular inflammation, a recent study by Yuan et al. utilized single-cell RNA sequencing to map the immune landscape of human AAA tissue. This analysis identified a novel transcriptionally distinct neutrophil subset characterized by high expression of membrane metalloendopeptidase (MME⁺). These MME⁺ neutrophils were significantly enriched in AAA tissues compared to healthy aortas and exhibited a gene signature associated with heightened inflammatory signaling, immune activation, and ECM remodeling. Notably, they expressed elevated levels of ADAM (a disintegrin and metalloproteinase) and ADAMTS (a disintegrin and metalloproteinase with thrombospondin motifs) proteases, which are critical to ECM degradation, indicating a mechanistic link between neutrophil-driven inflammation and the structural deterioration of the aortic wall. These findings position MME⁺ neutrophils as key pathogenic drivers of human AAA, capable of modulating both immune and stromal components of disease progression and presenting them as possible targets for therapeutic intervention [59]. These findings emphasize the importance of dissecting neutrophil diversity in cardiovascular diseases. Therapeutically targeting pathogenic subsets while preserving neutrophils with essential host defense functions may open new avenues for more precise immunomodulation in vascular pathology.

## Neutrophils are key players in the pathogenesis of AAA

**Fig. 3:**
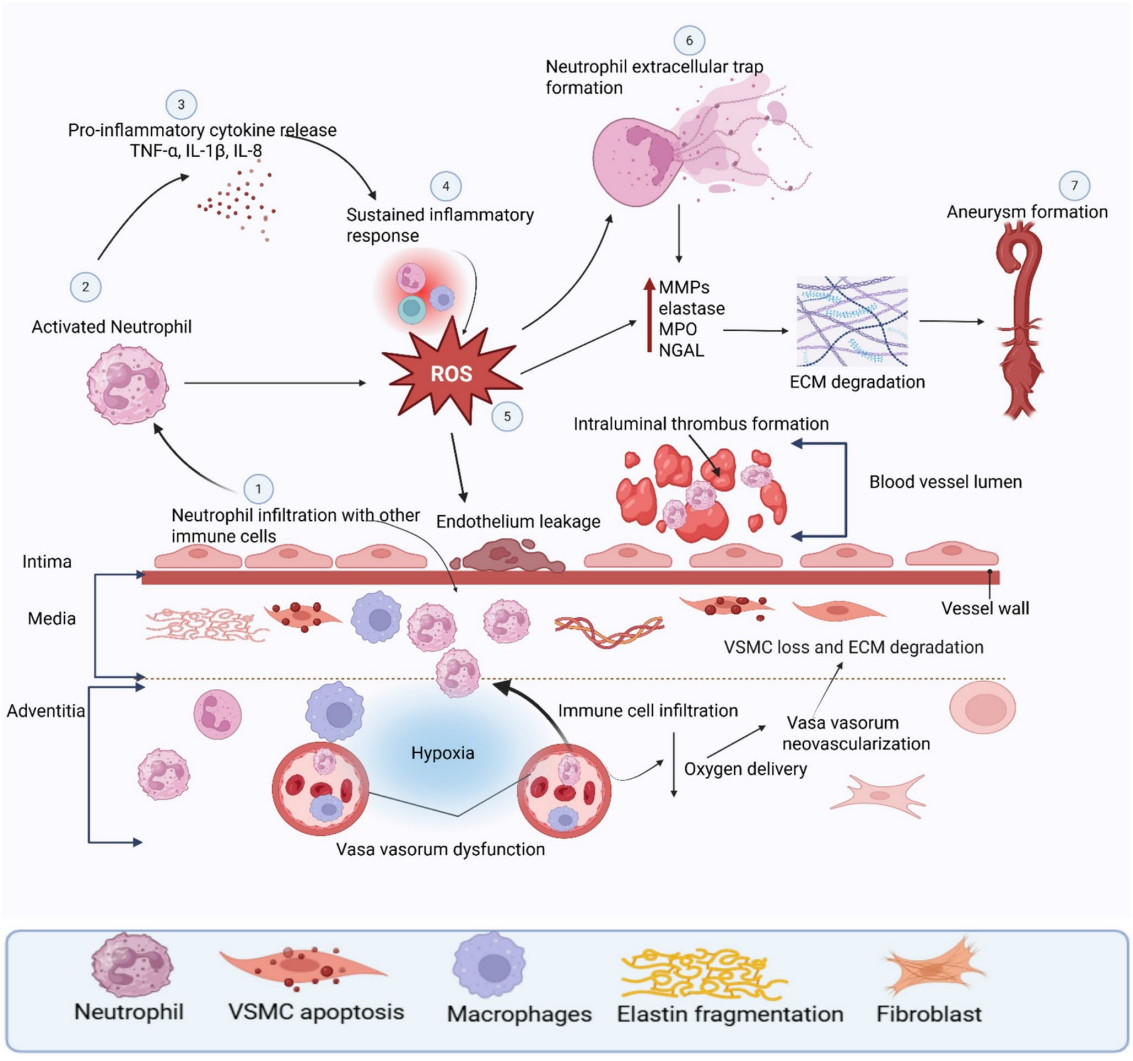
This schematic illustrates how neutrophils contribute to AAA pathogenesis through their recruitment to the aortic wall and intraluminal thrombus, where they release proteolytic enzymes, generate ROS, and form NETs. These processes promote extracellular matrix degradation, vascular remodeling, and chronic inflammation, facilitating aneurysm development and progression

Neutrophils have emerged as critical contributors to AAA pathogenesis, participating in various pathological processes, including oxidative stress, ECM degradation, adventitial inflammation, and the formation of ILT (Fig. 3) [60]. Upon activation, neutrophils secrete various effector molecules, including ROS, proteolytic enzymes such as MMPs, and granule proteins like MPO, NE, cathepsin G, and proteinase-3 [61]. These effector molecules collectively contribute to the degradation of structural proteins such as elastin and collagen in the aortic wall, compromising the integrity of the extracellular matrix [62]. Additionally, neutrophils amplify the recruitment of other immune cells through chemokine and cytokine production [51]. This feed-forward loop perpetuates chronic inflammation, weakening of the aortic wall, loss of VSMC, and significant thinning of the medial layer. Mouse models of AAA have consistently shown that neutrophils are among the first immune cells to infiltrate the aortic wall following aneurysm induction, underscoring their upstream role in the disease pathogenesis. Accumulating evidence implicates the vasa vasorum as critical conduits for inflammatory cell infiltration, particularly neutrophils, into the adventitial layer [63]. The vasa vasorum are microvessels within the adventitia that supply oxygen and nutrients to the aortic wall. Dysfunction of the adventitial vasa vasorum compromises mural perfusion, leading to hypoxia-driven inflammation, matrix metalloproteinase activation, and elastin–collagen degradation that weaken the vessel wall [64]. Tanaka et al. demonstrated that experimental hypoperfusion of the aortic vasa vasorum in rats induces wall hypoxia and recapitulates key features of human AAA, including medial degeneration and inflammation [64]. In a separate study, it was demonstrated that patients undergoing AAA repair exhibit intimal hyperplasia and luminal stenosis of the adventitial vasa vasorum in the aneurysm sac [63]. This remodeling is associated with reduced wall perfusion, decreased Heme B signal, and increased hypoxia-inducible factor 1-alpha (HIF-1α) expression, linking vasa vasorum dysfunction to wall ischemia [63]. Histological and molecular analyses in human and murine studies have established the ILT as a significant site of neutrophil accumulation in AAA [65]. The ILT, which forms in areas of low shear stress along the endothelial lining of the aneurysmal sac, provides a hypoxic and inflammatory niche that sustains neutrophil recruitment and activation. Neutrophils localize predominantly to the luminal layer of the ILT, releasing MMPs, including MMP-9, MMP-2, and MMP-8, which contribute to extracellular matrix degradation and weakening of the aortic wall [66]. Studies have detected neutrophil-specific markers such as CD66b, NGAL, and proteinase-3 concentrated in the innermost ILT regions and adventitia of human AAA tissue [67]. Further evidence from human studies shows that the ILT’s luminal layer exhibits vigorous chemotactic activity, primarily driven by neutrophil-recruiting cytokines such as interleukin-8 (IL-8) and platelet-derived chemokine RANTES. These chemokines are localized to platelet-neutrophil aggregates in the ILT, emphasizing a crosstalk between hemostatic and immune mechanisms that sustains neutrophil retention in the thrombus microenvironment [68]. Emerging evidence suggests that modulating neutrophil activation or recruitment may provide a therapeutic opportunity to suppress AAA progression. In a clinical trial, two weeks of doxycycline treatment before AAA repair significantly reduced mRNA levels of MMP-3 and MMP-25, along with protein levels of neutrophil-associated proteases MMP-8 and MMP-9. Immunohistochemistry confirmed a 75% reduction in neutrophil infiltration in the aneurysmal wall, indicating that doxycycline can suppress neutrophil-mediated matrix degradation [69]. In a seminal study using a murine elastase perfusion model, Eliason et al. demonstrated that antibody-mediated depletion of neutrophils prior to elastase infusion significantly attenuated aneurysm formation [70]. Only 8% of neutrophil-depleted mice developed AAA compared to 67% of control mice. Intriguingly, no significant differences in MMP-2 or MMP-9 levels were observed between the two groups. He et al. identified Family With Sequence Similarity 3, Member D (FAM3D) as a chemokine markedly upregulated in human AAA and elastase- and CaPO_4_-induced mouse aneurysmal aortas, where it orchestrates neutrophil recruitment and aneurysm progression [71]. Flow cytometry analysis revealed that *Fam3d*⁻^/^⁻ mice exhibited significantly reduced neutrophil infiltration in the aorta during early AAA formation compared to wild-type controls. Intraperitoneal administration of the FAM3D-neutralizing antibody 6D7 markedly suppressed elastase-induced AAA development and neutrophil recruitment. In addition, mitochondrial dysfunction in VSMCs is a well-recognized feature of AAA, characterized by impaired oxidative phosphorylation, increased mitochondrial reactive oxygen species production, and enhanced susceptibility to apoptosis [72]. This dysfunction is thought to arise, at least in part, as a downstream consequence of oxidative stress and ECM damage, linking the activity of neutrophils and other inflammatory cells to disease pathogenesis. Liu et al. demonstrated that VSMC mitochondrial dysfunction progressively worsens during AAA development, and that administration of synthetic high-density lipoprotein (sHDL) restores mitochondrial homeostasis and attenuates aneurysm formation in both angiotensin II (AngII) and porcine pancreatic elastase (PPE)–induced mouse models [73]. Consistently, reanalysis of microarray data from human AAA tissues revealed a significant downregulation of mitochondrial-related genes and VSMC contractile genes compared with normal aortic tissues [73]. Interestingly, neutrophils have been well-established as key regulators of fibroblast behavior, acting as orchestrators of tissue inflammation and remodeling across multiple inflammatory diseases. Lin et al. recently demonstrated that NETs activate fibroblasts, leading to smooth muscle cell proliferation via Wnt5a-mediated signaling. Inhibition of this pathway attenuates vascular remodeling in an experimental model of lower limb ischemia [74]. However, direct evidence of neutrophil–fibroblast crosstalk in AAA remains limited, highlighting a critical gap in our understanding of adventitial remodeling mechanisms. The multifaceted involvement of neutrophils, ranging from early recruitment and cytokine signaling to matrix degradation and redox imbalance, positions them as critical effector cells in AAA. Therapeutic strategies targeting neutrophil recruitment, activation, or effector functions may thus offer viable avenues for halting or slowing down aneurysm progression.

## Neutrophil recruitment to the AAA wall

Neutrophil recruitment to the aneurysm wall is a complex multi-step process that involves various molecular and cellular mechanisms. Neutrophils are among the first responders to inflammation. Neutrophils express adhesion molecules like selectins and integrins on their surface, which interact with complementary molecules on endothelial cells. During AAA development, the endothelium becomes activated by pro-inflammatory stimuli, circulating risk factors and disturbed flow, leading to the upregulation of adhesion molecules such as intercellular adhesion molecule-1 (ICAM-1), vascular cell adhesion molecule-1 (VCAM-1), and selectins (E- and P-selectin). These molecules facilitate neutrophil rolling, firm adhesion, and transmigration across the endothelium into the aortic wall [75].

The initial interaction is mediated by selectins, primarily E-selectin and P-selectin, which are expressed on activated endothelial cells in response to inflammatory stimuli such as TNF-α or IL-1β [76]. The binding of selectins to their ligands on neutrophils allows them to tether and roll along the vessel wall [77]. Following the rolling phase, adhesion is mediated by β2 integrins particularly lymphocyte function-associated antigen-1 (LFA-1, CD11a/CD18), and macrophage antigen-1 (Mac-1, CD11b/CD18) which are activated by chemokines such as CXCL1, CXCL2, and CXCL8 through chemokine receptor–dependent integrin activation [78, 79]. These integrins bind to ICAM 1 and 2 on the endothelium, stabilizing the interaction and promoting firm adhesion [80]. Trans endothelial migration is then facilitated by these interactions with platelet endothelial cell adhesion molecule-1 (PECAM-1), junctional adhesion molecules (JAMs), and CD99, guiding neutrophils through the endothelial junctions into the aortic wall [68]. In vitro studies show that cholesterol enrichment in human neutrophils facilitates adhesion to activated endothelial cells and promotes trans-endothelial migration across EC monolayers [81]. A previous study examined human neutrophil adhesion to fibronectin following stimulation with angiotensin II and aldosterone in vitro. Neither stimulus altered neutrophil attachment suggesting that angiotensin II and aldosterone do not directly enhance integrin-mediated adhesion in vitro. However, aldosterone specifically induced the release of hydroxylysine, MMP-9, and cathepsin G, suggesting a role in extracellular matrix remodeling and enhanced neutrophil recruitment to the aortic wall [82]. Nobiletti et al. reported that neutrophil-specific deletion of Krev interaction-trapped-1 (KRIT1) showed reduced adhesion and spreading on fibronectin, accompanied by increased migration [83]. In contrast, adhesion to and migration on ICAM-1 remained unaffected, highlighting a selective role for KRIT1 in neutrophil–matrix interactions.

L-selectin has been identified as a critical regulator of early neutrophil recruitment. Hannawa et al. showed that L-selectin expression is significantly upregulated in the aorta after elastase infusion [84]. This increase correlates with enhanced neutrophil infiltration, particularly on day seven, when aneurysm formation becomes more prevalent. Genetic deletion of L-selectin in the elastase-induced AAA model resulted in a marked reduction in both immune cell accumulation and the size of the aneurysm, suggesting a vital role for L-selectin-mediated leukocyte rolling in the initial inflammatory cascade of AAA. Houard et al. demonstrated that the ILT is a highly active inflammatory niche, where neutrophils and platelets colocalize and provide a rich source of chemotactic factors such as RANTES, platelet factor 4, and interleukin 8 (IL-8) [68]. In vitro blockade of RANTES and IL-8 significantly decreases neutrophil migration, indicating that a neutrophil–platelet interaction axis contributes to maintaining ILT-driven inflammation.

The alternative complement pathway has emerged as a crucial regulator of neutrophil recruitment in the pathogenesis of elastase-induced AAA. Depletion of complement activity using cobra venom factor significantly reduced aneurysm formation, highlighting the critical role of this pathway in AAA pathogenesis. Both C3a and C5a anaphylatoxins independently promote sustained neutrophil infiltration, enhancing the local inflammatory environment. These findings suggest that complement activation not only initiates but also perpetuates the destructive vascular remodeling in AAA [85]. Pagano et al. demonstrated that dipeptidyl peptidase I (DPPI), a key activator of neutrophil granule-associated serine proteases, critically modulates the chemokine milieu necessary for neutrophil recruitment during aneurysm formation [86]. DPPI-deficient mice exhibited markedly reduced expression of the neutrophil chemoattractant CXCL2 and were significantly protected from elastase-induced AAA. In summary, the recruitment of neutrophils through a specific cascade of adhesion molecules facilitates their infiltration into the aortic wall, where they contribute to AAA.

## Neutrophil protease release and ECM degradation

### Neutrophil gelatinase-associated lipocalin (NGAL)

NGAL, primarily secreted by neutrophils, has been increasingly recognized for its role in AAA progression [87]. Neutrophils at sites of inflammation promote the formation of NGAL/MMP-9 complexes, which protect MMP-9 from proteolytic degradation and enhance its enzymatic activity. These complexes are abundant throughout the ILT, particularly in the luminal layer, facilitating ECM degradation in the adjacent vessel walls and contributing to aneurysm expansion [88]. Although elevated plasma levels of NGAL/MMP-9 complexes have been observed in AAA patients, they do not correlate with maximal aortic diameter or ILT thickness, suggesting NGAL serves more as an indicator of thrombus activity rather than a predictor of aneurysm growth [89]. Clinically, blood NGAL levels are significantly elevated in patients with ruptured AAA compared to non-ruptured cases, with rupture sites showing markedly higher NGAL expression in aneurysmal tissue than in non-dilated aortas [90]. Additionally, both serum and urine NGAL have emerged as promising early indicators of acute kidney injury in AAA patients undergoing open surgical repair, highlighting its potential as dual biomarker for vascular instability and postoperative renal complications [91]. Experimental murine models have provided compelling evidence for NGAL’s role in AAA pathogenesis. Both genetic deletion and antibody-mediated inhibition of NGAL in the elastase-induced murine model significantly suppressed aneurysm growth, primarily by reducing neutrophil infiltration, limiting ECM degradation, and decreasing MMP activity, while preserving VSMC integrity [92]. Activation of the c-Jun N-terminal kinase (JNK) pathway has been shown to induce the expression of both NGAL and MMP-9 within the aortic wall [93]. Inhibition of the JNK pathway effectively suppressed AAA formation in two independent murine models, highlighting NGAL as a promising marker of AAA size and progression.

### Neutrophil elastase

Neutrophil Elastase (NE), a serine protease abundantly expressed in neutrophils, has emerged as a key contributor to ECM remodeling and inflammatory signaling in AAA. NE proteolytic activity is further amplified through the activation of MMPs (MMP-2, MMP-3, and MMP-9), while concurrently suppressing ECM preservation by inactivating their endogenous inhibitor, tissue inhibitor of metalloproteinase-1 (TIMP-1) [94]. Upregulated NE function has been demonstrated in human AAA tissue and plasma, correlating with the pathological progression and intensity [94, 95]. Clinical studies strongly implicate NE in AAA pathogenesis, with markedly elevated circulating NE levels consistently observed in the blood of AAA patients compared to non-AAA controls [96]. Notably, cigarette smoking, an established risk factor for AAA, enhances NE release and its proteolytic activity [97]. Elevated NE activity correlates with increased aneurysm size, ILT burden, and mechanical stress, positioning NE as a potential biomarker for disease progression. Experimental studies have further validated the functional importance of NE in AAA development. NE-knockout mice exhibited reduced aneurysm formation in both AngII and CaCl₂-induced models [98]. In line with these findings, pharmacological inhibition of NE using a novel oral inhibitor (AZD9668) in a rat model significantly reduced AAA diameters, neutrophil activation markers, and elastase activity in conditioned media from aneurysmal tissue [99].

### Myeloperoxidase

MPO is a heme-containing enzyme predominantly stored in the azurophilic granules of neutrophils. Upon activation, neutrophils release MPO into the phagolysosomal and extracellular space, generating reactive oxidants. While MPO is protective in microbial killing, excessive neutrophil-derived MPO activity promotes oxidative stress, inflammation, and extracellular matrix damage in the vasculature, contributing to AAA progression. A study investigating the role of MPO in AAA found that patients exhibited nearly a twofold increase in plasma MPO levels and more than an elevenfold elevation in aortic tissue MPO concentrations compared to healthy controls [100]. This marked increase in circulating and tissue-localized MPO levels reflected heightened neutrophil activation and oxidative stress within the aneurysmal wall [100]. The study further identified a strong positive correlation between MPO levels and maximal aneurysm diameter, suggesting that MPO serves as a biomarker of disease activity and may contribute directly to AAA progression through sustained vascular inflammation and ECM degradation [100]. Another study found that elevated baseline MPO levels are significantly linked to fast AAA progression, independent of the initial aortic diameter. In predicting aneurysm growth, MPO demonstrated a prognostic value with 80% sensitivity and 59% specificity, highlighting its potential utility as a biomarker for monitoring AAA advancement [101].

### Matrix metalloproteinases

MMPs are a family of zinc-dependent endopeptidases central to orchestrating ECM remodeling. Neutrophils, as a predominant cellular source of MMP-8 and MMP-9, play a critical role in mediating ECM remodeling through the secretion of these proteolytic enzymes [102]. MMP-9 has been identified as a key effector, with its expression significantly elevated in human AAA tissues compared to non-aneurysmal aortas, underscoring its prominent role in aneurysm pathogenesis [103]. Compelling evidence from a meta-analysis of case-control studies revealed significantly elevated circulating MMP-9 levels in AAA patients compared to controls, indicating a strong association between elevated MMP-9 levels and the presence of AAA [104]. MMP-9 expression has been found to correlate with larger aneurysm diameters (> 5 cm), suggesting its involvement in advanced stages of disease [103]. Locally, MMP-9 concentrations in the aneurysmal wall correlate with ILT thickness [105]. MMP-8 and MMP-9 levels are significantly elevated at rupture sites compared to non-ruptured regions of the same aneurysm [106]. Mouse studies have established that genetic ablation of MMP-9 significantly attenuates AAA formation in the elastase and CaCl_2_-induced models, highlighting MMP-9 as a critical mediator of aneurysmal remodeling [107, 108]. However, the specific contribution of neutrophil-derived MMP-9 in this process remains unexplored, representing a critical gap in our understanding of AAA pathogenesis.

## Neutrophils and oxidative stress

**Fig. 4:**
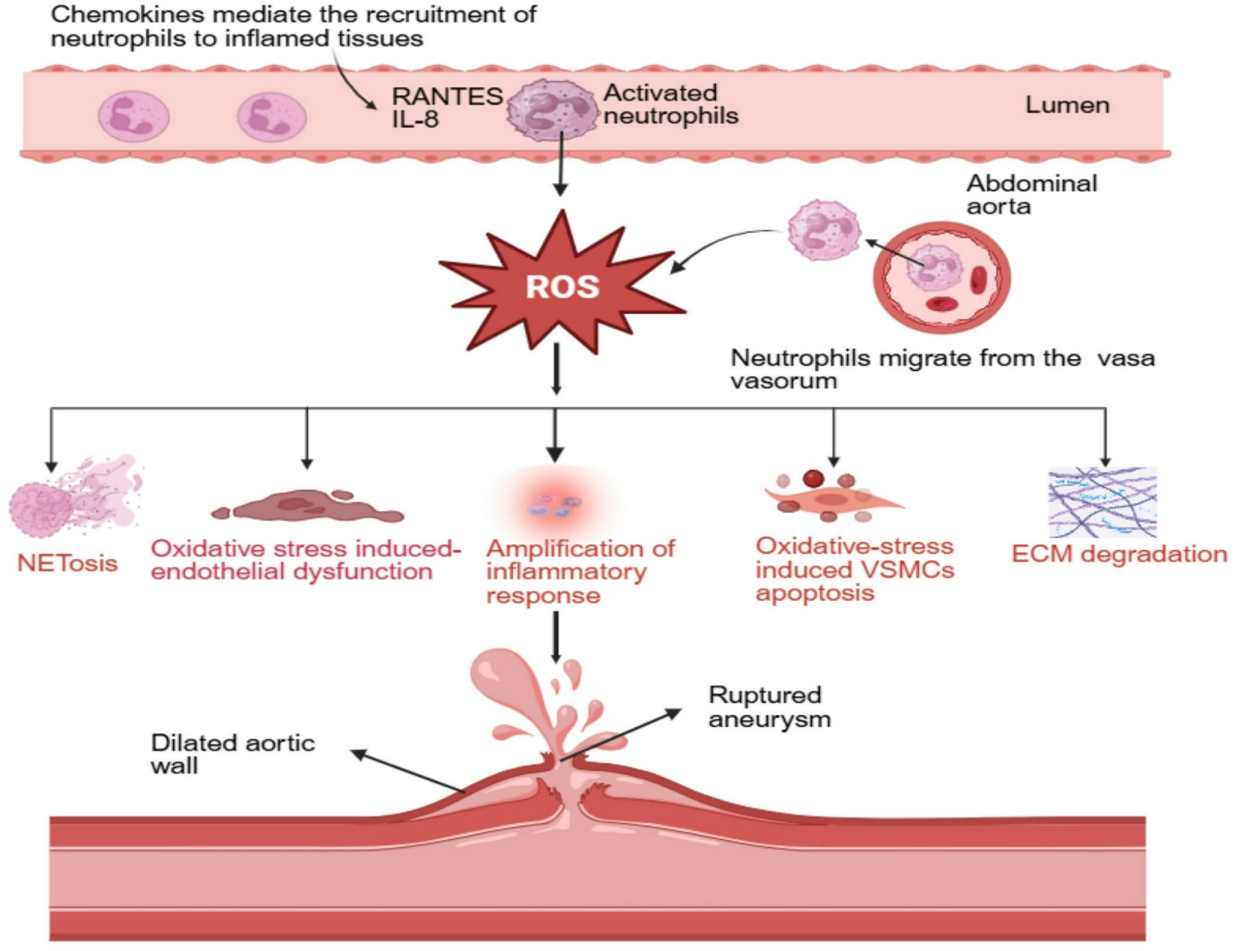
Neutrophils recruited to the abdominal aorta generate ROS, driving vascular inflammation in AAA. Excess ROS promotes NETosis, endothelial dysfunction, amplified inflammatory signaling, VSMC apoptosis, and extracellular matrix degradation. Together, these processes weaken and dilate the aortic wall, ultimately contributing to aneurysm progression and rupture

A critical factor in the pathogenesis of aortic aneurysms is oxidative stress resulting from the excessive generation of free radicals and ROS within the aortic wall. ROS are highly reactive oxygen intermediates produced during cellular metabolism or host stress responses. At controlled levels, ROS serve as critical secondary messengers in redox signaling, immune modulation, and antimicrobial defense. The NADPH oxidase (NOX) enzyme family includes NOX1, NOX2, NOX3, NOX4, and NOX5, and the dual oxidases DUOX1 and DUOX2. The NOX enzymes comprise membrane-bound and cytosolic subunits that specialize in generating ROS by transferring electrons across the membrane, from cytosolic NADPH to molecular oxygen [109]. The NOX enzymes are rapidly activated by various external stimuli, enabling the localized and robust production of superoxide anion (O_2_^•–^) in response to physiological and pathological signals. NOX4 is constitutively active and produces primarily hydrogen peroxide (H_2_O_2_). In neutrophils, the primary source of superoxide anion is NADPH oxidase 2 (NOX2). NOX2, a 91 kDa transmembrane glycoprotein, functions as the catalytic core of the NOX complex. This multi-subunit enzyme system consists of the membrane-bound homodimer flavocytochrome b_558_ (gp91^*phox*^/NOX2 and p22^*phox*^) and cytosolic regulatory subunits (p47^*phox*^, p67^*phox*^, p40^*phox*^ ), along with the GTPase Rac (Table 1) [110]. The p22^*phox*^ subunit is essential for the structural and functional stability of NOX2 in the plasma membrane, where it facilitates heme incorporation into the catalytic core, a prerequisite for O_2_^•–^ generation [111]. Its C-terminal region contains a proline-rich region (PRR) that serves as a critical docking interface for the SH3 (SRC Homology 3) domains of cytosolic subunits, particularly p47^*phox*^ , and p40^*phox*^ , thereby initiating the early steps of NOX2 assembly [112]. Under basal conditions, p47^*phox*^ and p40^*phox*^ adopt autoinhibitory conformations that mask their interaction domains, preventing spontaneous activation of the oxidase complex [113]. Upon neutrophil stimulation, p47^*phox*^ undergoes multisite phosphorylation on specific serine residues by kinases such as protein kinase C (PKC) and mitogen-activated protein kinases (MAPKs) [114]. These phosphorylation events trigger a conformational shift that exposes its SH3 domains and polybasic region, enabling simultaneous interaction with the PRR of p22^*phox*^ and phosphoinositide-rich domains in the plasma membrane. This membrane targeting phosphorylated p47^*phox*^ promotes the recruitment of its cytosolic partners, p67^*phox*^ and p40^*phox*^, forming the functional NOX2 complex. Concurrently, Rac2, a Rho family GTPase abundantly expressed in neutrophils, becomes activated through GDP-GTP exchange, dissociates from its GDP dissociation inhibitor (GDI), and translocates to the membrane. Membrane anchoring of Rac2 is mediated by its polybasic domain and prenylated C-terminus, ensuring spatial proximity to the assembling complex. GTP-bound Rac2 directly interacts with p67^*phox*^, a critical step that enhances its binding to NOX2 and promotes conformational changes necessary for electron transfer. This interaction is thought to regulate, in concert with p67^*phox*^, the catalytic function of NOX2, enabling the transfer of electrons from NADPH in the cytosol to molecular oxygen on the extracellular side or within the phagosome, resulting in the production of O_2_^•–^ [115]. The paper by Gao et al. emphasizes that infiltrating inflammatory cells, notably neutrophils and macrophages, are major sources of ROS, which inflict direct damage on VSMCs contributing to their phenotypic modulation or their apoptosis (Fig. 4) [116]. ROS also play a significant role in degrading the ECM by activating matrix MMPs such as MMP-2 and MMP-9, accelerating the breakdown of essential structural proteins such as elastin and collagen. Neutrophil-derived enzymes and oxidative mediators are key drivers of vascular remodeling and tissue damage in AAA. Among these, MPO has emerged as a central mediator of neutrophil-induced oxidative stress. MPO catalyzes the formation of hypochlorous acid, a potent oxidant that modifies proteins, lipids, and extracellular matrix components. Kim et al. demonstrated that genetic deletion of MPO significantly reduced AAA formation in both AngII-infused apolipoprotein E-deficient mice and elastase-perfused *C57BL/6* mice [117]. Similarly, taurine, an MPO-derived oxidant scavenger, attenuated AAA development across multiple models while decreasing oxidative stress markers such as aortic peroxidase activity and protein-bound dityrosine. MPO deficiency and taurine administration suppressed macrophage infiltration, matrix metalloproteinase activation, and elastin degradation. In a separate study, proteomic profiling analysis revealed reduced catalase activity and increased H_2_O_2_ and MPO levels in circulating neutrophils from AAA patients, indicating a shift toward a pro-oxidative phenotype [118]. Recently, Mehrkens et al. demonstrated that MPO neutralization attenuates thoracic aortic aneurysm formation in a mouse model of Marfan disease [119]. In CaCl₂-induced murine models of AAA, apocynin robustly attenuated aneurysm formation, accompanied by marked reductions in ROS production and aortic MMP‑2 and MMP‑9 expression, highlighting the central role of NOX-dependent oxidative stress in driving aneurysm progression [120]. Siu et al. demonstrated that NOX2 and other NOX isoforms deletion in angiotensin II–infused hph‑1 mice markedly attenuate AAA incidence and aortic dilation, suppresses superoxide production, restores eNOS coupling, and enhances nitric oxide and tetrahydrobiopterin (BH₄) bioavailability, identifying NOX-derived ROS as critical upstream drivers of aneurysm formation [121]. Interestingly, Kigawa et al. reported that global NOX2 knockout in angiotensin II–infused *Ldlr*^-/^^-^ mice paradoxically exacerbates AAA development. Despite lowering ROS, NOX2 deficiency drives M1 macrophage polarization with elevated IL‑1β and MMP9/12, an effect reversed by IL‑1β neutralization, underscoring the context-dependent immunoregulatory role of NOX2 in AAA pathogenesis [122]. This paradox highlights a fundamental gap in our understanding of NOX2 biology in aneurysm formation. Selective pharmacologic NOX2 inhibition has not been evaluated in AAA, and the role of neutrophil-specific NOX2 remains unexplored, limiting insight into how neutrophil-derived ROS orchestrate vascular inflammation and matrix degradation. Addressing this gap is essential to define whether NOX2 represents a viable and cell-specific therapeutic target in AAA.

**Table 1:** NOX expression and subunit composition in human and mouse neutrophils

Components	Human neutrophils	Mouse neutrophils	Primary function	Key interactions	References
NOX2 (gp91^*phox*^)	Highly expressed	Highly expressed	Catalytic core of NADPH oxidase in phagocytic ROS production.	Interacts with all the subunits	[123–126]
p22^*phox*^	Expressed	Expressed	Forms a membrane-bound heterodimer with NOX2.	gp91^*phox*^/NOX2	[124, 127]
* Cytosolic subunits*					
p47^*phox*^	Expressed	Expressed	It is an adaptor protein that initiates NOX2 activation by coordinating cytosolic complex assembly and membrane translocation via phosphorylation and PI(3,4)P₂ binding.	p22^*phox*^ (via SH3 domains), PI(3,4)P2 (via PX domain)	[126, 127]
p67^*phox*^	Expressed	Expressed	It is essential for NOX2 activation, facilitating electron transfer from NADPH to FAD and enabling superoxide anion production.	p47^*phox*^, Rac	[126, 127]
p40^*phox*^	Expressed	Expressed	Modulates NOX2 activation by targeting membranes through its PX domain and stabilizing the complex via interaction with p67^phox^.	p67^*phox*^, PI3P (via PX domain)	[126, 127]
Rac2 (small GTPase)	Highly expressed	Highly expressed	Regulates NOX2 activity; critical for assembly	p67^*phox*^	[126, 127]
* Other Nox isoforms*					
NOX1/NOX4/NOX5	Not significantly expressed	Low/Not detected	Limited or no role in neutrophils		[124, 125]

## Neutrophil-derived cytokines and chemokines

Neutrophil-associated cytokines contribute substantially to the inflammatory milieu of AAA. In vitro studies have been instrumental in uncovering the ability of neutrophils to produce a broad range of cytokines and chemokines, even though they generate fewer cytokine molecules per cell than monocytes or lymphocytes [30]. LPS, IFNγ, and bacterial peptides stimulate neutrophil expression of chemokines including CXCL1, CXCL2, and CCL3 [128]. Neutrophil-derived chemokines have been linked to the recruitment of additional neutrophils and monocytes, amplifying the inflammatory milieu and vascular remodeling in AAA [129]. Studies have shown that stimulation of neutrophils with IL-1β and TNFα can secrete matrix-degrading enzymes and pro-inflammatory mediators relevant to aortic wall remodeling [130, 131]. Neutrophil recruitment to the AAA site is primarily driven by IL-8, which is highly concentrated at the luminal surface of the ILT [132], where neutrophil infiltration is the most prominent. ILT-derived IL-8 concentrations have been reported to be approximately four times higher than those released by the aortic wall (media and adventitia), indicating a dominant role of the ILT as a source of neutrophil-attracting chemokines in AAA [68]. Animal studies have also clarified the significance of neutrophil-derived cytokines in AAA development. For example, in a murine model of elastase-induced AAA, inhibition of CXCL1 and its receptor CXCR2 significantly reduced aneurysm formation, implicating neutrophil chemotaxis and activation as central to aneurysm progression [133]. Infiltrating neutrophils serve as a key source of IL-1β, a cytokine that induces NET formation and contributes to AAA pathogenesis [134]. Experimental studies have demonstrated significantly elevated IL-1β mRNA and protein levels in murine abdominal aortas following AAA induction, with both genetic deletion and pharmacologic blockade of IL-1β signaling effectively reducing aneurysm formation in two distinct mouse models [135]. Similarly, the inhibition of TNF-α via tumor necrosis factor binding protein conferred protection against AAA development in an elastase-induced rat model [136]. Chemokines such as CCL3 and CXCL1 are not only upregulated in aneurysmal tissues but have also been linked to leukocyte accumulation and adventitial inflammation [137]. These observations suggest that neutrophil-derived cytokines operate at the innate and adaptive immunity interface, orchestrating a complex inflammatory cascade that compromises aortic wall integrity. Future therapies may benefit from selectively targeting neutrophil cytokine production or trafficking to mitigate aneurysm progression without broadly suppressing host defense.

## Neutrophil extracellular traps (NETs) in AAA pathogenesis

NETs are web-like chromatin structures released by activated neutrophils, composed of decondensed nuclear DNA and a repertoire of bioactive proteins from nuclear, cytoplasmic, and membrane origin. These include histones, high mobility group box 1 protein (HMGB1), lamin B, MPO, NE, cathepsin G, proteinase 3, MMP9, LL-37, lactoferrin, complement components, and tissue factor [138–140]. Importantly, NETs serve as platforms for activating and processing proinflammatory cytokines [141], thereby sustaining and amplifying inflammatory cascades. AAA is marked by persistent neutrophilic infiltration, progressive extracellular matrix breakdown, and loss of VSMCs. The proteolytic arsenal carried by NETs, including MMP9, NE, and cathepsin G, actively targets key structural components such as elastin and collagen, weakening the aortic wall. Simultaneously, NET-associated DAMPs and cytokines amplify local inflammatory circuits and drive continued recruitment of immune cells, fueling a self-perpetuating cycle of vascular injury and remodeling. NET formation is primarily regulated by intracellular ROS and calcium signaling. The NOX-dependent pathway, activated via the MAPK/ERK cascade, induces ROS production [142]. This triggers the release and nuclear translocation of MPO and NE, promoting chromatin decondensation and membrane rupture [143]. Alternatively, calcium influx activates peptidyl arginine deiminase 4 (PADI4), which citrullinates histones, facilitating chromatin relaxation and DNA release independent of NOX2 [144]. In vitro, these pathways are commonly studied using phorbol myristate acetate (PMA) to stimulate NOX2-dependent NETosis or calcium ionophores to induce the PADI4-mediated pathway [145]. The detection of NETs in both human AAA tissue and experimental models further supports their pathogenic role, highlighting them as key mediators of aortic wall degeneration in AAA. Neutrophil NOX2 activity is linked to the regulation of NET formation. Administration of NOX2-derived metabolites, including H₂O₂ and hypochlorous acid, has been shown to induce NETosis [146, 147]. Consistently, neutrophils isolated from chronic granulomatous disease (CGD) patients (caused by mutations in NOX2 or its subunits) display defective NET formation, whereas gene therapy restoring NOX2 activity re-establishes their normal NETotic activity [148]. The first evidence implicating NETs in AAA pathogenesis was reported by Delbosc et al. in 2011, who identified NET accumulation in the ILT and adventitia of AAA tissues, along with elevated circulating levels of cell-free DNA and MPO-DNA complexes in AAA patients compared to healthy controls [149]. Yan et al. demonstrated that NETs promote AAA development by triggering plasmacytoid dendritic cells (pDCs) to produce type I interferons, thereby intensifying local inflammation in experimental mouse models [150]. They observed co-localization of NET-derived LL-37 with pDCs in human AAA tissues, a feature absent in healthy aortas. The study concluded that degrading NETs with DNase I markedly reduced elastase-induced AAA formation in mice, underscoring the role of NETosis in early disease progression. NETs were detected in human AAA tissue co-localized with IL-1β. In vitro, IL-1β induced NETosis in neutrophils via ceramide synthase 6-dependent C16-ceramide production. They further demonstrated that NETs emerged as early as day 3 post-induction in a murine elastase-induced AAA model. Genetic deletion of IL-1β conferred protection against aneurysm formation, whereas adoptive transfer of wild-type neutrophils restored aneurysm susceptibility, underscoring the pathogenic role of IL-1β–driven neutrophil activity in early AAA development [134]. Resolvin D1, a specialized pro-resolving lipid mediator, significantly reduced aneurysm progression in both elastase- and angiotensin II–induced murine AAA models by suppressing IL-1β, MMP-2, and NET markers (CitH3, NE), while increasing the anti-inflammatory cytokine IL-10 [151]. A subsequent study reported abundant NETs in excised aneurysmal wall and thrombus tissue, along with elevated plasma levels of citrullinated H3 (CitH3) in AAA patients compared to healthy controls. CitH3 levels normalized following surgical repair and demonstrated prognostic value for disease progression [152]. These findings suggest that NETs accumulate locally within the aneurysm wall, contribute to local and systemic inflammatory responses, and serve as potential biomarkers for AAA activity and progression [153]. Wei et al. demonstrated that PADI4-dependent NET formation promotes apoptosis in VSMCs through p38 and JNK signaling, driving AAA rupture. Pharmacological inhibition of NETosis with YW3-56 preserved aortic wall integrity and markedly reduced rupture risk in the an AngII–induced model [154]. In parallel, NETs have also been linked to ferroptosis of VSMCs. Qi et al. demonstrated that NETs disrupt mitochondrial glutathione metabolism by destabilizing the SLC25A11 transporter, triggering VSMC ferroptosis and accelerating aneurysmal degeneration [155]. Emerging evidence highlights a key role for NETs in driving AAA by promoting a synthetic and proinflammatory phenotype in VSMCs through inhibition of the Hippo-YAP pathway. Elevated NET components in serum and tissue correlated with worse clinical outcomes in AAA patients. Padi4 or Yap knockout mice showed reduced VSMC phenotypic switching and were protected against AAA development in the AngII–induced AAA model [156]. Ibrahim et al. reported that NET formation is most pronounced during the early stages of AAA development, rather than during later progression, in both elastase- and AngII–induced mouse models [157]. They further demonstrated that upstream inhibition of NET formation is more effective than downstream NET inactivation in limiting AAA progression. Notably, NET blockade was particularly beneficial in mice that developed intramural thrombus and was associated with reduced vascular remodeling, highlighting the therapeutic relevance of early and targeted NET intervention in AAA. Current AAA treatments lack precision therapies targeting the underlying inflammatory processes, particularly neutrophil-driven NET formation. Recently, innovative nanoparticle-based approaches have shown promise in addressing this gap. Hu et al. demonstrated that luminol-conjugated α-cyclodextrin nanoparticles (LaCD NP) effectively target neutrophil-mediated inflammation and NET formation in AAA [158]. In a CaCl₂-induced rat model, these nanoparticles selectively accumulated in aneurysmal tissue, leading to reductions in aortic expansion, elastin degradation, and calcification. They further showed that LaCD NP markedly reduced NET formation, curtailing NET-driven inflammation and the detrimental processes contributing to AAA progression. Complementing the role of nanomedicine, Zhang et al. demonstrated that metallothionein 1 (MT1), previously identified as a negative regulator of NETosis, promotes AAA progression by enhancing NET formation and inducing VSMC dysfunction [159]. MT1 expression was significantly elevated and correlated with NET formation in both human and murine AAA tissues. They demonstrated that silencing MT1 with glycoRNA-coated nanoparticles (siMT1) markedly reduced neutrophil infiltration, NET formation, and AAA progression in a porcine pancreatic elastase (PPE)-induced model. More recently, integrative bioinformatics and machine learning approaches identified six key NETosis-related genes (DUSP26, FCN1, MTHFD2, GPRC5C, SEMA4A, and CCR7) with strong diagnostic potential for predicting AAA progression. In vitro studies showed that silencing CCR7 and MTHFD2 effectively blocked angiotensin II-induced phenotypic switching, dysfunction, and senescence in VSMCs, underscoring their pivotal role in AAA development [160]. PI3Kγ was identified as an upstream regulator of NET formation and aortic inflammation in a PPE-induced AAA model. PI3Kγ deficiency suppressed AAA development by inhibiting the noncanonical pyroptosis pathway, reducing NETosis. This regulatory effect was mediated through the cAMP/PKA signaling axis, revealing a key mechanistic link between PI3Kγ, pyroptosis, and NET-driven AAA progression [161]. Michalska et al. reported elevated levels of NET-associated markers, dsDNA, ssDNA, and citH3 following stent-graft implantation in thoracoabdominal aneurysm patients [162]. While histone concentrations normalized postoperatively, persistently high extracellular DNA levels indicated ongoing NET reorganization and a sustained inflammatory environment. These findings suggest that NETs may influence not only aneurysm pathogenesis but also post-interventional vascular remodeling and long-term clinical outcomes, reinforcing their relevance as therapeutic targets in AAA.

## Neutrophil-targeted interventions in experimental AAA

Neutrophil-targeted therapeutic approaches in AAA focus on blocking neutrophil recruitment and inflammatory activation, NETosis, protease release, and downstream oxidative and inflammatory cascades that weaken the aortic wall (Table 2). A large body of preclinical work implicates NETosis and ROS as drivers of matrix degradation, VSMC death, vascular remodeling and inflammation in AAA [154]. Inhibition of neutrophil-mediated vascular inflammation and adverse aortic remodeling in the AAA wall is, therefore, an attractive therapeutic strategy. Targeting intracellular signaling pathways that regulate NETosis is another promising therapeutic strategy. Recent work showed that PI3Kγ contributes to NET formation and that PI3Kγ inhibition or genetic deficiency attenuates NETosis, decreases aortic wall inflammation, and limits AAA development in mice, highlighting opportunities to repurpose selective kinase inhibitors [161]. Neutralizing neutrophil proteases and limiting ECM degradation remain attractive therapeutic targets for AAA. Broad-spectrum MMP inhibition with doxycycline reduced MMP levels and neutrophil-associated proteolytic activity in both preclinical and early clinical studies and provided a rationale for further investigation [163]. Neutrophil elastase inhibitors (e.g., AZD9668) showed benefits in animal models of AAA but lack definitive human data [164]. These anti-protease strategies may be most effective when combined with other interventions that reduce neutrophil influx or NETosis. More recently, nanomedicine and cell-based delivery systems have provided innovative strategies to modulate neutrophil activity in AAA by delivering anti-NET and antioxidant agents directly to the aneurysmal wall. Chen et al. showed that mesenchymal stem cell–derived extracellular vesicles (MSC-EVs) attenuate AAA progression in AngII mice model by suppressing NET formation and redirecting neutrophil death toward apoptosis. MSC-EVs reduced aortic dilation, elastin degradation, and NET-associated vascular smooth muscle cell injury, highlighting NETosis inhibition as a therapeutic strategy in AAA [165]. Translation of this experimental data into effective clinical treatments for patients remains challenging because of the heterogeneity of human AAA, complex disease etiology, variable contributing mechanisms and risk factors, and potential concerns about long-term pan-neutrophil inhibition strategies. Future progress will depend on rational combination therapies that target multiple aspects of neutrophil activation to achieve effective and safe modulation of neutrophil-driven inflammation.

**Table 2:** Summary of neutrophil-targeted therapeutic strategies in AAA. The table highlights key neutrophil-targeted therapeutic approaches in experimental AAA, grouped by their biological mechanisms. These include strategies targeting NET formation, neutrophil recruitment, neutrophil protease release, and inflammatory cytokine production

Target/Pathway	Therapeutic approach	Model	Effect on AAA
NETosis/NET formation	PAD4 inhibitor (YW3-56) PI3Kγ inhibition DNase 1	Ang II-infused in ApoE^−/−^ mice Ang II-induced AAA Elastase-induced AAA	Decreased NETs reduced aortic diameter, less VSMC apoptosis, less elastin degradation, and reduced AAA rupture [155]. Inhibition of PI3Kγ suppressed non-canonical pyroptosis. Less NET formation and less aortic wall inflammation [162]. DNase 1 dismantles NETs and suppresses AAA formation [151].
Neutrophil recruitment to the aortic wall	Genetic deletion of L-selectin In vitro blockade of RANTES and IL-8 signaling Complement depletion using cobra venom factor (CVF)	Elastase-induced AAA Human ILT analyses, ex vivo chemotaxis assays Elastase-induced AAA	L-selectin knockout mice showed significantly reduced neutrophil infiltration and smaller aneurysms [85]. ILT is an inflammatory niche enriched in neutrophils and platelets. Blocking RANTES and IL-8 significantly reduced neutrophil migration [69]. CVF-mediated complement depletion significantly reduced aneurysm formation [86].
Neutrophil proteases	Genetic deletion of dipeptidyl peptidase I (DPPI) Neutrophil elastase inhibitor (Silvelestat/ONO-5046)	Elastase-induced AAA Ang II-induced AAA in ApoE^−/^^−^ mice	DPPI-deficient mice showed markedly reduced CXCL2 expression and were protected from elastase-induced AAA [151]. Reduced elastase activity, decreased neutrophil infiltration, reduced MMP-9, and attenuated aneurysm growth [165].
Inflammatory cytokine induced-NETosis	IL-1β blockade (IL-1RA), NETosis inhibitor (CI-amidine)	Human neutrophils in vitro Elastase-induced AAA	IL-1RA reduced NETosis in vitro. IL-1β knockout mice had fewer neutrophils in the aorta and were protected from AAA, and CI-amidine also reduced AAA [135].

## Conclusion and future studies

Neutrophils have emerged as critical drivers of AAA development and progression. A key mechanism by which they contribute to this pathology is through the secretion of ROS, proteolytic enzymes, and granule proteins that degrade structural components such as elastin and collagen within the aortic wall [61]. Despite compelling evidence that neutrophils contribute to AAA pathology, no selective neutrophil-targeted therapies have been tested in human AAA clinical trials, representing a clear and urgent gap between preclinical insight and clinical translation. Future research must move beyond descriptive associations to mechanistically dissect the complex inflammatory programs through which neutrophils drive AAA initiation and progression. A priority area is defining neutrophil heterogeneity within human AAA tissues using high-resolution single-cell and spatial transcriptomic approaches. While emerging data suggests the presence of pathogenic neutrophil subsets enriched for proteolytic, redox, and NET-associated signatures, their developmental origin, longevity within the intraluminal thrombus, and interactions with macrophages, platelets, and VSMC remain incompletely understood. Mapping these subsets and their communication networks will be essential for developing subset-specific therapies that preserve systemic host defense. Circulating neutrophil-based indices, particularly the neutrophil-to-lymphocyte ratio (NLR), have emerged as accessible biomarkers of systemic inflammation with prognostic relevance in AAA. Elevated NLR is independently associated with increased short and long-term mortality following both endovascular and open AAA repair [166, 167]. Moreover, higher NLR correlates with aneurysm rupture risk and increased 30-day mortality, with thresholds such as NLR > 5 identifying high-risk patients [168]. Collectively, these findings support the concept that blood neutrophil counts and NLR may serve as simple, cost-effective biomarkers for risk stratification and assessment of disease severity in AAA, further reinforcing the clinical relevance of neutrophil-driven inflammation. Critically, the next wave of therapeutic exploration should prioritize targeted delivery strategies and the use of primary human cells and human AAA tissue. Systemic neutrophil suppression carries infection risk, but localized delivery of anti-inflammatory agents using nanoparticles may allow effective suppression of neutrophil-driven inflammation with minimal systemic toxicity. Collectively, future work integrating cellular profiling, mechanistic redox–NET studies, biomarker development, and localized therapeutic platforms will be essential for translating neutrophil-targeted interventions into clinically meaningful strategies to inhibit AAA progression and prevent rupture.

## Data Availability

No datasets were generated or analysed during the current study.

## Abbreviations

AAA: Abdominal Aortic Aneurysm
ADAM: A disintegrin and metalloproteinase
ADAMTS: A disintegrin and metalloproteinase with thrombospondin motifs
CSF3: Colony-Stimulating Factor 3
CMPs: Common Myeloid Progenitors
ECM: Extracellular Matrix
fMLP: N-formyl-methionyl-leucyl-phenylalanine
G-CSF: Granulocyte Colony-Stimulating Factor
GMPs: Granulocyte-Monocyte Progenitors
HSCs: Hematopoietic Stem Cells
HDNs: High Density Neutrophils
HIV: Human Immunodeficiency Virus
ILT: Intraluminal Thrombus
IFN: Interferon
LMPPs: Lymphoid-Primed Multipotent Progenitors
LDNs: Low-Density Neutrophils
LDGs: Low-Density Granulocytes
MPO: Myeloperoxidase
MMPs: Matrix Metalloproteinases
MME: Membrane Metalloendopeptidase
MAPKs: Mitogen-Activated Protein Kinases
NADPH: Nicotinamide Adenine Dinucleotide Phosphate
NADPH oxidase: NOX
NE: Neutrophil Elastase
PMA: Phorbol-Myristate-Acetate
ROS: Reactive Oxygen Species
